# Trends of dental caries in permanent teeth among 12-year-old Chinese children: evidence from five consecutive national surveys between 1995 and 2014

**DOI:** 10.1186/s12903-021-01814-7

**Published:** 2021-09-23

**Authors:** Zhen Hu, Xiaojin Yan, Yi Song, Shang Ma, Jun Ma, Guangrong Zhu

**Affiliations:** 1 Institute of Child and Adolescent Health, School of Public Health, Peking University, No. 38 Xueyuan Rd, Haidian District, Beijing, 100191 China; 2 Division of Prevention and Community Health, National Center for Cardiovascular Disease, Fuwai Hospital, Peking Union Medical College & Chinese Academy of Medical Sciences, Mentougou District, Beijing, China; 3 Institute of Population Research, Peking University, Beijing, China; 4 School of Health Humanities, Peking University, Beijing, China

**Keywords:** Permanent teeth, Prevalence, DMFT, Caries filling ratio, Children, China

## Abstract

**Background:**

Dental caries have a serious impact on general health and well-being; however, there is a lack of relevant data on the development trends of dental caries in permanent teeth among 12-year-old children in China. We aim to assess long-term trends of dental caries in permanent teeth among 12-year-old children in China and identify the susceptible subgroups based on five consecutive national surveys from 1995 to 2014.

**Methods:**

A total of 88 972 subjects were extracted from five consecutive national surveys (1995, 2000, 2005, 2010, 2014). Standardized dental examinations were conducted and the oral health status of each subject was recorded. The prevalence of Decayed, Missing and Filled teeth (DMF%), mean Decayed, Missing, Filled teeth score (DMFT) and Caries Filling Ratio (CFR) were used as measurement indicators. Cochran–Armitage trend test was used to evaluate the trends in DMF% and CFR, and multivariate linear regression was used to evaluate the trends in DMFT.

**Results:**

A V-shaped fluctuating upward trend in DMF% during 1995–2014 was observed (Z =  − 13.124, *P* < 0.001), and the DMF% in 1995–2014 was 21.1%, 15.9%, 16.2%, 21.9% and 24.3%. The trend in DMFT was approximately consistent with DMF% (*β* = 0.057, *P* < 0.001), but the downward volatility appeared in 2014. The DMFT in 1995–2014 was 0.38, 0.28, 0.31, 0.66 and 0.54. A continuously fluctuant trend in CFR was observed during past two decades (Z = 1.927, *P* > 0.05), and the CFR in 1995–2014 was 17.4%, 22.8%, 19.3%, 23.4% and 15.6%. The DMF% and DMFT of rural children had a larger absolute increase than that of urban children during 1995–2014 (DMF%-urban: Z =  − 0.242, *P* > 0.05; DMF%-rural: Z =  − 19.036, *P* < 0.001; DMFT-urban: *β* = 0.035, *P* < 0.001, DMFT-rural: *β* = 0.077, *P* < 0.001). The DMF% and DMFT in girls were higher than that in boys at each survey year *(P* < 0.001). CFR of urban children was higher than that of rural children at each survey year *(P* < 0.001).

**Conclusions:**

Over the past 20 years, DMFT and DMF% of 12-year-old children in China presented V-shaped fluctuant upward trends, with a decline trend from 1995 to 2000 and an upward trend from 2000 to 2014. CFR had no significant improvement. The rural children and girls are the more vulnerable groups in the development of dental caries and need to pay priority. Our study supports the continuation of policies to improve children’ oral health.

**Supplementary Information:**

The online version contains supplementary material available at 10.1186/s12903-021-01814-7.

## Background

Dental caries are a major public health problem as one of the most widespread non-communicable diseases (NCD) worldwide [1]. Together with tumor and hypertension, dental caries are listed by the World Health Organization (WHO) as the three major non-communicable diseases that need to be mainly prevented and treated [2]. Everyone is at risk of dental caries, while children have the highest. WHO recommended the age of 12 as the target age for global dental caries detection and the prevalence of Decayed, Missing and Filled teeth (DMF%), mean Decayed, Missing, Filled teeth score (DMFT) and Caries Filling Ratio (CFR) as the measurement indicators of dental caries in permanent teeth [3]. Globally, the data of 1995–1996 showed that DMFT of Chinese children aged 12 was 1.0 ~ 0.9, which was at a very low level [4]. However, the trend of dental caries generally emerged a relatively fluctuating trend and was inconsistent in various countries [5]. A previous Chinese study reported a trend of permanent dental caries in 12-year-olds from 1991 to 2005 [6]. However, the data they use was old and outdated so that it needs to be updated urgently.

Numerous studies have shown that dental caries had different distributions across areas and gender. Urban–rural differences in dental caries exist but with controversial findings, for example, one survey from North-Eastern Italy indicated that the prevalence of decay is lower in an urban context among schoolchildren [7]. Another survey conducted in India manifested that children had a higher risk of dental caries if they lived in urban area [8]. So did the gender disparities in dental caries. In Guwahati city, the boys exhibited higher caries prevalence than the girls [9], while girls were at higher risk than boys in Indonesia [10]. However, vulnerable population of children with dental caries in China has been less researched.

Previous studies demonstrated that during the past two decades, Chinese children have experienced a rapid lifestyle transition [11], per capita output of sugar crops dramatically increased from 65.90 kg in 1995 to 88.61 kg in 2014 [12]. The consumption proportion of sugar-containing food in girls was higher than that of boys [13]. However, few representative data is available to estimate the national trends of dental caries in permanent teeth of 12-year-old Chinese children or identify the susceptible subgroups. Therefore, the nationally representative Chinese National Survey on Students’ Constitution and Health (CNSSCH) provided a unique opportunity for us to fill the gaps. In this study, we aim to assess national trends of dental caries in permanent teeth among 12-year-old Chinese children and identify the susceptible subgroups based on five consecutive national surveys between 1995 and 2014.

## Methods

This study was approved by the Medical Research Ethics Committee of the Peking University Health Science Center (IRB00001052-18002). Informed consent was obtained verbally because written consent was difficult to obtain in the national surveys.

### Study design

Consecutive cross-sectional studies were respectively conducted in 1995, 2000, 2005, 2010 and 2014 in China.

### Setting

The CNSSCH is a national survey, which has been conducted every five years since 1985, jointly launched by the Ministry of Education, the Ministry of Health, the Ministry of Science and Technology, the State of Nation Affairs, and the State Sports General Administration of People’s Republic of China. The objective of the CNSSCH studies is to grasp the Chinese students’ constitution and health status and development trend, promote local and school to fully implement the Party’s education policy in the new era, and scientifically evaluate the efficiencies of school physical education, hygiene and health education. It provides the basis for making the development plan and scientifically carrying out the work of school physical education, hygiene and health education. The test items include 24 indicators from 4 aspects, including body shape, physiological function, physical fitness and health status. Our report used the data of caries in 12-year-old children from the CNSSCH study. The CNSSCH used a multistage stratified cluster sampling design and has maintained consistent approaches to sampling and assessment across the different survey years, as previously described [14–16]. Briefly, all the subjects were primary and high school students aged 7–18 years randomly selected from 30 of the 31 mainland provinces, excluding Tibet. In each province, survey sites and schools were randomly selected from three socioeconomic slices (“upper”, “moderate” and “low”). In each survey school, the class was taken as the unit for random cluster sampling, stratified by grade. The number of classes selected should meet the minimum sample size. It was determined that sample sizes could be reduced without greatly sacrificing the survey’s statistical analyses power. The subjects were divided into four categories: urban, rural, male and female, with a minimum sample size of 50 people for each section, each category and each age group.

### Participants and study size

Between 1995 and 2014, 1 084 649 children and adolescents aged 7–18 years participated in the CNSSCH, of those, 88 972 participants aged 12 were included in our study as the analysis sample.

### Data sources/measurement

A total of 60 to 150 oral professionals were involved in the clinical examination process at each survey year, all of them were required to pass a training course for the investigation. They performed oral examinations in sufficient lighting using a surface reflection mirror and a No.5 explorer in 1995 and 2000 survey year, with disposable oral instrument box replaced since 2005. Standardized dental examinations were conducted in a uniform sequence and Decayed, Missing and Filled teeth condition of permanent teeth were recorded. Thirty mainland provinces were analysed in our study, and they were divided into three regions: the east region, the central region and the west region according to the geographical standard division from the National Bureau of Statistics of China [17].

Diagnostic criteria for dental caries were as following [18, 19]:

The lesion in fissure or the smooth surface of the tooth has a soft base, with underlying damaged enamel or wall softening, also including temporary fillings (such as zinc oxide) in the teeth. Caries include fissure caries and smooth surface caries (teeth with caries on the adjacent, buccal and lingual surfaces).

‘Decayed tooth’ (DT) designates a tooth in which a cavity could clearly be seen or a lesion could be felt with an explorer in a pit or fissure, or on a smooth surface.

‘Missing tooth’ (MT) designates a tooth that had been lost or extracted for dental caries not at replacement age. Third molars were excluded.

‘Filled tooth’ (FT) designates a tooth with one or more permanent restorations and no cavity anywhere on the tooth.

### Variables

(1) DMF%: the percentage (%) of the persons who had decayed, missing and filled in permanent teeth among all participants examined. When calculating, the total numbers of persons who had decayed, missing, filled in permanent teeth were used as the numerator, and the number of all participants examined were used as the denominator, which was expressed as a percentage (%).

(2) DMFT: the mean number of caries (D + M + F) among all participants examined. When calculating, the total teeth numbers of decayed, missing and filled in permanent teeth were used as the numerator, and the number of all participants examined were used as the denominator, which was expressed as mean ± standard deviation (‾X ± S).

(3) CFR: the ratio of the total teeth number of filling to the total teeth number of decayed, missing and filled in permanent teeth among all participants examined. When calculating, the total teeth numbers of filling in permanent teeth were used as the numerator, and the total teeth numbers of decayed, missing and filled in permanent teeth were used as the denominator, which was expressed as a percentage (%).

### Bias

The CNSSCH has maintained consistent approaches to sampling and assessment across the different survey years. Participants with missing data or biologically implausible values were excluded from the study. Dental caries measurements and diagnosis in the CNSSCH followed a standardized procedure and were conducted by trained and qualified oral professionals. The principles underlying the methods and instruments were the same at each survey site.

### Statistical methods

Categorical variables were characterized by frequencies and percentages, and continuous variables were characterized as mean ± standard deviation. Chi-square test was used to compare the gender differences of DMF% and CFR in overall and subgroups of each survey year, and *t*-test was used to compare the gender differences of DMFT in overall and subgroups of each survey year. In addition, Cochran–Armitage trend test was utilized to evaluate the trends of DMF% and CFR in overall and subgroups, and Multivariate linear regression was utilized to evaluate the trends of DMFT in overall and subgroups. Two-sided *P* < 0.05 was considered to be statistically significant. All statistical analyses were conducted with R version 4.0.2 software (R Foundation for Statistical Computing, Vienna, Austria) and SPSS, version 20.0 (IBM, Armonk, New York).

## Results

### Participants and descriptive data

The study sample consisted of 88,772 children, with 17,305, 17,609, 18,213, 17,852 and 17,793 participants from 1995 to 2000, respectively. The distribution of participants by area (rural 50.8%) and gender (girls 50.0%) was basically balanced. The demographic characteristics of the participants are provided in Table 1.

**Table 1:** Demographic characteristics of the participants

	Boys	Girls	Total
Overall	44,404 (50.0)	44,368 (50.0)	88,772 (100)
**1995**			
Total	8720 (50.4)	8585 (49.6)	17,305 (100.0)
Area			
Urban	4393 (50.0)	4393 (50.0)	8786 (50.8)
Rural	4327 (50.8)	4192 (49.2)	8519 (49.2)
Region			
East	3385 (50.0)	3382 (50.0)	6767 (39.1)
Central	2537 (50.0)	2537 (50.0)	5074 (29.3)
West	2798 (51.2)	2666 (48.8)	5464 (31.6)
**2000**			
Total	8748 (49.7)	8861 (50.3)	17,609 (100.0)
Area			
Urban	4414 (49.8)	4446 (50.2)	8860 (50.3)
Rural	4334 (49.5)	4415 (50.5)	8749 (49.7)
Region			
East	3614 (48.9)	3783 (51.1)	7397 (42.0)
Central	2281 (49.8)	2297 (50.2)	4578 (26.0)
West	2853 (50.6)	2781 (49.4)	5634 (32.0)
**2005**			
Total	9121 (50.1)	9092 (49.9)	18,213 (100.0)
Area			
Urban	4573 (50.1)	4549 (49.9)	9122 (50.1)
Rural	4548 (50.0)	4543 (50.0)	9091 (49.9)
Region			
East	3517 (50.7)	3416 (49.3)	6933 (38.1)
Central	2190 (49.1)	2272 (50.9)	4462 (24.5)
West	3414 (50.1)	3404 (49.9)	6818 (37.4)
**2010**			
Total	8913 (49.9)	8939 (50.1)	17,852 (100.0)
Area			
Urban	4452 (49.9)	4473 (50.1)	8925 (50.0)
Rural	4461 (50.0)	4466 (50.0)	8927 (50.0)
Region			
East	3266 (50.0)	3272 (50.0)	6538 (36.6)
Central	2361 (49.9)	2373 (50.1)	4734 (26.5)
West	3286 (49.9)	3294 (50.1)	6580 (36.9)
**2014**			
Total	8902 (50.0)	8891 (50.0)	17,793 (100.0)
Area			
Urban	4453 (50.0)	4445 (50.0)	8898 (50.0)
Rural	4449 (50.0)	4446 (50.0)	8895 (50.0)
Region			
East	3282 (49.9)	3294 (50.1)	6576 (37.0)
Central	2393 (50.0)	2390 (50.0)	4783 (26.9)
West	3227 (50.2)	3207 (49.8)	6434 (36.2)

### Outcome data

#### The trend of DMF% in overall and subgroups

**Fig. 1:**
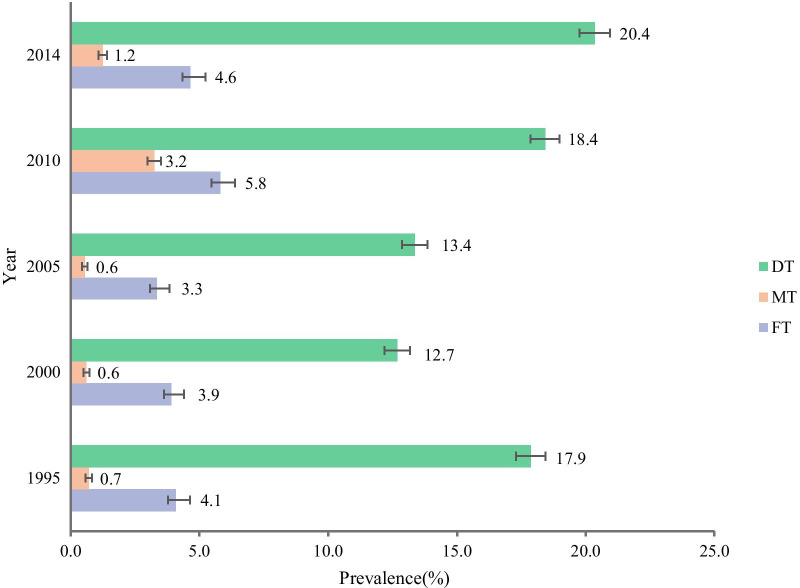
Trend of DT%, MT%, FT% in Chinese 12-year-old children from1995 to 2014. *DT* The percentage (%) of the persons who had decayed in permanent teeth among all participants examined; *MT* The percentage (%) of the persons who had missing in permanent teeth among all participants examined; *FT* The percentage (%) of the persons who had filled in permanent teeth among all participants examined

Nationwide, we observed a V-shaped fluctuating upward trend in DMF% during 1995–2014 period (Z =  − 13.124, *P* < 0.001), and the DMF% decreased from 21.1% in 1995 to 15.9% in 2000, and then consistently increased from 16.2% in 2005 to 24.3% in 2014 (Table 2, Additional file 1: Table 1). The DT% in 1995–2014 was 17.9%, 12.7%, 13.4%, 18.4% and 20.4%, the MT% was 0.7%, 0.6%, 0.6%, 3.2% and 1.2%, the FT% was 4.1%, 3.9%, 3.3%, 5.8% and 4.6% (Fig. 1, Additional file 2: Table 2). When further stratified by DT%, MT% and FT%, the trends of DT% showed similar pattern with the total sample. During 1995–2014, DMF% of rural children had a dramatically larger absolute increase than that of urban counterparts (urban: Z =  − 0.242, *P* > 0.05; rural: Z =  − 19.036, *P* < 0.001). In 2014, DMF% of rural children (24.7%) even exceeded that of their urban peers (23.8%). Meanwhile, DMF% of girls were higher than that of boys in all survey years and almost all subgroups (*P* < 0.001). Except in 2010, the DMF% in the east was higher than that in the west and central regions (*P* < 0.001) (Table 2, Additional file 1: Table 1).

**Table 2:** DMF% in 12-year-old Chinese children by gender (%, 95%CI)

	Overall	Boys	Girls	Chi-square-test *P* value
**1995**				
Total	21.1 (20.5–21.7)	18.9 (18.1–19.7)	23.4 (22.5–24.3)	< 0.001
Area				
Urban	26.0 (25.0–26.9)	22.9 (21.7–24.1)	29.0 (27.7–30.4)	< 0.001
Rural	16.1 (15.3–16.9)	14.8 (13.8–15.9)	17.5 (16.3–18.6)	< 0.001
Region				
East	27.4 (26.3–28.5)	24.0 (22.5–25.4)	30.8 (29.3–32.4)	< 0.001
Central	17.3 (16.2–18.3)	14.8 (13.4–16.2)	19.7 (18.2–21.3)	< 0.001
West	16.9 (15.9–17.9)	16.5 (15.1–17.9)	17.4 (16.0–18.8)	0.360
**2000**				
Total	15.9 (15.3–16.4)	14.0 (13.3–14.8)	17.7 (16.9–18.5)	< 0.001
Area				
Urban	18.0 (17.2–18.8)	16.4 (15.3–17.5)	19.5 (18.3–20.7)	< 0.001
Rural	13.8 (13.0–14.5)	11.6 (10.7–12.6)	15.8 (14.8–16.9)	< 0.001
Region				
East	22.7 (21.8–23.7)	20.1 (18.8–21.4)	25.3 (23.9–26.6)	< 0.001
Central	9.7 (8.8–10.5)	9.1 (7.9–10.3)	10.2 (9.0–11.5)	0.187
West	12.0 (11.1–12.8)	10.4 (9.3–11.5)	13.6 (12.3–14.8)	< 0.001
**2005**				
Total	16.2 (15.7–16.8)	13.9 (13.2–14.6)	18.6 (17.8–19.4)	< 0.001
Area				
Urban	16.8 (16.1–17.6)	14.2 (13.2–15.2)	19.5 (18.3–20.6)	< 0.001
Rural	15.7 (14.9–16.4)	13.6 (12.6–14.6)	17.8 (16.7–18.9)	< 0.001
Region				
East	20.0 (19.0–20.9)	16.5 (15.3–17.7)	23.6 (22.1–25.0)	< 0.001
Central	13.9 (12.9–14.9)	12.7 (11.3–14.1)	15.0 (13.5–16.4)	0.032
West	14.0 (13.2–14.8)	11.9 (10.8–13.0)	16.1 (14.9–17.3)	< 0.001
**2010**				
Total	21.9 (21.3–22.5)	19.2 (18.4–20.0)	24.6 (23.7–25.5)	< 0.001
Area				
Urban	22.5 (21.7–23.4)	19.8 (18.6–21.0)	25.3 (24.0–26.5)	< 0.001
Rural	21.3 (20.4–22.1)	18.7 (17.5–19.8)	23.9 (22.6–25.1)	< 0.001
Region				
East	20.4 (19.4–21.4)	16.5 (15.2–17.8)	24.3 (22.8–25.8)	< 0.001
Central	20.7 (19.6–21.9)	18.7 (17.1–20.3)	22.7 (21.0–24.4)	< 0.001
West	24.2 (23.2–25.3)	22.3 (20.9–23.7)	26.2 (24.7–27.7)	< 0.001
**2014**				
Total	24.3 (23.6–24.9)	20.3 (19.5–21.2)	28.2 (27.3–29.1)	< 0.001
Area				
Urban	23.8 (22.9–24.7)	19.7 (18.6–20.9)	27.9 (26.5–29.2)	< 0.001
Rural	24.7 (23.8–25.6)	20.9 (19.8–22.1)	28.5 (27.2–29.9)	< 0.001
Region				
East	28.6 (27.5–29.7)	24.0 (22.5–25.4)	33.2 (31.6–34.8)	< 0.001
Central	21.4 (20.2–22.6)	17.8 (16.2–19.3)	25.1 (23.3–26.8)	< 0.001
West	22.0 (20.9–23.0)	18.6 (17.2–19.9)	25.4 (23.9–26.9)	< 0.001

#### The trend of DMFT in overall and subgroups

**Fig. 2:**
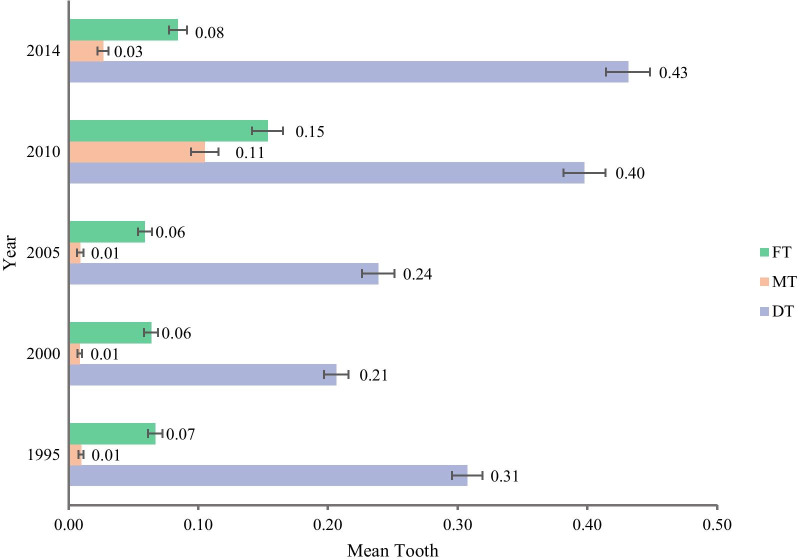
Trend of DT, MT, FT in Chinese 12-year-old children from1995 to 2014. *DT* The mean number of decayed teeth among all participants examined; *MT* The mean number of missing teeth among all participants examined; *FT* The mean number of filled teeth among all participants examined

A fluctuant upward trend of the DMFT was discovered during 1995–2014 (*β* = 0.057, *P* < 0.001), with a decrease from 0.38 in 1995 to 0.28 in 2000 and an increase to 0.66 in 2010, then a fell to 0.54 in 2014. Trend in DMFT was generally consistent with that in DMF%. However, the downward volatility appeared in 2014 (Table 3, Additional file 1: Table 1). The DT in 1995–2014 was 0.31, 0.21, 0.24, 0.40 and 0.43, the MT was 0.01, 0.01, 0.01, 0.11 and 0.03, the FT was 0.07, 0.06, 0.06, 0.15 and 0.08 (Fig. 2, Additional file 2: Table 2). During 1995–2014, DMFT of rural children had a remarkably greater absolute increase than that of urban counterparts (urban: *β* = 0.035, *P* < 0.001, rural: *β* = 0.077, *P* < 0.001). DMFT of rural children (0.54) got almost same level with their urban counterparts (0.54) in 2014. Meanwhile, DMFT of girls were higher than that of boys in all survey year and almost all subgroups (*P* < 0.001). The regional differences in DMFT were consistent with that in DMF% (*P* < 0.001) (Table 3, Additional file 1: Table 1).

**Table 3:** DMFT in 12-year-old Chinese children by gender (means, SD)

	Overall	Boys	Girls	*t*-test P value
**1995**				
Total	0.38 (0.90)	0.33 (0.81)	0.44 (0.98)	< 0.001
Area				
Urban	0.48 (1.00)	0.40 (0.88)	0.56 (1.10)	< 0.001
Rural	0.29 (0.78)	0.25 (0.73)	0.32 (0.83)	< 0.001
Region				
East	0.52 (1.05)	0.43 (0.92)	0.62 (1.17)	< 0.001
Central	0.30 (0.80)	0.26 (0.75)	0.35 (0.84)	< 0.001
West	0.28 (0.75)	0.26 (0.69)	0.30 (0.80)	0.054
**2000**				
Total	0.28 (0.76)	0.23 (0.68)	0.32 (0.84)	< 0.001
Area				
Urban	0.31 (0.78)	0.27 (0.71)	0.35 (0.85)	< 0.001
Rural	0.25 (0.75)	0.20 (0.64)	0.30 (0.83)	< 0.001
Region				
East	0.42 (0.95)	0.35 (0.84)	0.49 (1.04)	< 0.001
Central	0.14 (0.49)	0.13 (0.47)	0.15 (0.51)	0.137
West	0.20 (0.63)	0.16 (0.56)	0.24 (0.70)	< 0.001
**2005**				
Total	0.31 (0.97)	0.25 (0.84)	0.36 (1.09)	< 0.001
Area				
Urban	0.32 (1.02)	0.26 (0.81)	0.38 (1.19)	< 0.001
Rural	0.29 (0.92)	0.25 (0.86)	0.34 (0.98)	< 0.001
Region				
East	0.39 (1.15)	0.31 (0.96)	0.47 (1.32)	< 0.001
Central	0.26 (0.93)	0.23 (0.84)	0.28 (1.00)	0.040
West	0.25 (0.77)	0.21 (0.68)	0.30 (0.85)	< 0.001
**2010**				
Total	0.66 (2.13)	0.57 (1.95)	0.74 (2.28)	< 0.001
Area				
Urban	0.81 (2.65)	0.71 (2.42)	0.91 (2.86)	< 0.001
Rural	0.50 (1.40)	0.44 (1.32)	0.57 (1.48)	< 0.001
Region				
East	0.42 (1.03)	0.32 (0.92)	0.51 (1.11)	< 0.001
Central	0.39 (0.99)	0.34 (0.88)	0.44 (1.08)	< 0.001
West	1.09 (3.20)	0.99 (2.94)	1.18 (3.43)	0.018
**2014**				
Total	0.54 (1.30)	0.42 (1.08)	0.66 (1.48)	< 0.001
Area				
Urban	0.54 (1.27)	0.41 (1.06)	0.67 (1.45)	< 0.001
Rural	0.54 (1.33)	0.43 (1.10)	0.66 (1.51)	< 0.001
Region				
East	0.69 (1.50)	0.54 (1.27)	0.84 (1.69)	< 0.001
Central	0.46 (1.19)	0.35 (0.96)	0.57 (1.37)	< 0.001
West	0.46 (1.13)	0.36 (0.94)	0.56 (1.29)	< 0.001

#### The trend of CFR in overall and subgroups

A continuously fluctuant trend of the CFR during 1995–2014 (Z = 1.927, *P* > 0.05) was observed, with a decrease from 17.4% in 1995 to 22.8% in 2000 and an increase to 23.4% in 2010, then a decrease to 15.6% in 2014. CFR reached a bottom of 19.3% in 2005 and a summit of 23.4% in 2010. CFR of urban children is considerably higher than that of rural counterparts at each survey year (*P* < 0.001). There was no significant difference between boys and girls at each survey year (*P* > 0.05) (Table 4, Additional file 1: Table 1).

**Table 4:** CFR in 12-year-old Chinese children by gender (%, 95%CI)

	Overall	Boys	Girls	Chi-square-test *P* value
**1995**				
Total	17.4 (16.5–18.3)	17.1 (15.8–18.5)	17.6 (16.4–18.8)	0.641
Area				
Urban	23.8 (22.5–25.0)	23.8 (21.8–25.8)	23.8 (22.1–25.4)	0.983
Rural	6.4 (5.5–7.4)	6.5 (5.1–8.0)	6.3 (5.0–7.7)	0.848
Region				
East	23.4 (22.0–24.8)	23.5 (21.3–25.7)	23.4 (21.6–25.2)	0.920
Central	10.4 (8.9–11.9)	10.8 (8.5–13.2)	10.1 (8.1–12.1)	0.651
West	10.6 (9.0–12.1)	10.3 (8.1–12.5)	10.8 (8.6–12.9)	0.758
**2000**				
Total	22.8 (21.7–24.0)	22.1 (20.3–23.9)	23.3 (21.8–24.9)	0.333
Area				
Urban	33.3 (31.5–35.1)	32.1 (29.4–34.8)	34.2 (31.8–36.6)	0.247
Rural	9.7 (8.5–11.0)	8.4 (6.6–10.3)	10.5 (8.9–12.2)	0.107
Region				
East	26.0 (24.5–27.6)	25.4 (23.0–27.8)	26.5 (24.5–28.5)	0.507
Central	17.8 (14.9–20.8)	18.4 (14.0–22.8)	17.4 (13.4–21.3)	0.736
West	16.8 (14.6–18.9)	15.7 (12.4–19.0)	17.5 (14.6–20.4)	0.410
**2005**				
Total	19.3 (18.3–20.4)	20.2 (18.5–21.8)	18.7 (17.4–20.1)	0.176
Area				
Urban	25.6 (24.0–27.2)	25.8 (23.3–28.3)	25.4 (23.4–27.5)	0.796
Rural	12.4 (11.2–13.7)	14.2 (12.1–16.3)	11.2 (9.6–12.7)	0.020
Region				
East	26.8 (25.1–28.5)	28.5 (25.8–31.2)	25.7 (23.5–27.8)	0.103
Central	11.9 (10.0–13.8)	10.4 (7.7–13.1)	13.0 (10.4–15.6)	0.179
West	12.5 (10.9–14.1)	14.2 (11.7–16.8)	11.2 (9.3–13.2)	0.064
**2010**				
Total	23.4 (22.6–24.2)	23.8 (22.6–24.9)	23.1 (22.1–24.1)	0.677
Area				
Urban	27.8 (26.8–28.9)	28.5 (26.9–30.1)	27.3 (26.0–28.7)	0.278
Rural	16.3 (15.2–17.3)	16.1 (14.4–17.7)	16.4 (15.0–17.8)	0.767
Region				
East	26.9 (25.2–28.5)	28.9 (26.2–31.6)	25.6 (23.5–27.7)	0.060
Central	10.6 (9.2–12.0)	10.0 (7.9–12.1)	11.0 (9.1–12.9)	0.494
West	25.4 (24.4–26.4)	25.5 (24.0–27.0)	25.3 (23.9–26.6)	0.854
**2014**				
Total	15.6 (14.9–16.3)	14.9 (13.8–16.1)	16.0 (15.1–16.9)	0.161
Area				
Urban	18.7 (17.6–19.8)	18.7 (16.9–20.5)	18.7 (17.3–20.1)	0.998
Rural	12.5 (11.5–13.4)	11.3 (9.9–12.7)	13.2 (12.0–14.5)	0.043
Region				
East	23.1 (21.8–24.3)	22.4 (20.4–24.3)	23.5 (21.9–25.1)	0.377
Central	11.2 (9.8–12.5)	12.3 (10.1–14.6)	10.4 (8.8–12.1)	0.172
West	7.3 (6.3–8.2)	5.5 (4.1–6.8)	8.5 (7.2–9.8)	0.002

## Discussion

Globally, the prevalence of dental caries in 12-year-old children emerged a relatively fluctuant trend and showed controversial findings in various countries over the past 30 years. In Asian countries, DMFT had a decrease before 2006 and increase from 2006 to 2011 in Philippines, while in Japan, DMFT had a decreased yearly since 1975 [5]. In our study, we found that DMFT and DMF% of 12-year-old Chinese children presented a V-shaped fluctuant upward trend over the past 20 years, with a decline trend from 1995 to 2000 and a significantly continuous upward trend from 2000 to 2014, and reached the lowest level in 2000. Although compared with the results of European countries and the world [4, 20], both DMFT and DMF% were at relative low levels, the rising trend reminds us that the situation of caries in China is still not optimistic and faces great challenges.

From 1995 to 2000, the prevalence of dental caries in permanent teeth in 12-year-old Chinese children showed a decreasing trend, which may be related to the three oral health care strategies adopted in China during this period: (1) public oral health education; (2) pit and fissure sealing services; (3) fluoridation [21]. Regarding public oral health education, the ministry of education promulgated and stipulated a series of policies to promote oral health from the mid-1990s to the early 2000s [22, 23]. However, there was much less from 2000 to 2014. Moreover, the study found that after the reform of the basic education curriculum in 2001, school health education on dental caries was not well implemented, which may make it difficult to guarantee the timing and content of oral health education for children [24]. As for pit and fissure sealing service, China began to promote it nationally to prevent children’s dental caries in 1993 [25]. Dental sealant programs had been found to prevent up to 80% of tooth decay in the treated teeth [26]. Regarding using fluoride to prevent caries, nationwide pilot projects began to be carried out for fluoridated drinking water, salt, milk and toothpaste since 1991 [27]. Clinical trials have shown that fluoride can significantly reduce tooth decay [28]. However, due to safety concerns, the use of fluoride was not promoted after the end of the project, even though cariogenic foods and beverages were rapidly developed at the same time. Fluorinated toothpaste had been promoted and available nationwide since 1989, even though surveys show that its use is not popular [29].

From 2000 to 2014, the prevalence of dental caries in permanent teeth in 12-year-old Chinese children showed a significantly continuous upward trend, which may be related to changes in people’s dietary patterns and the rapid development of caries-causing foods and drinks, as well as the relative weakness of dental caries prevention and services. With the socioeconomic development [30], food choices became more diverse and the public consumption patterns shifted from high-fiber, low-sugar diets to high-sugar, high-energy diets, especially among children. Studies have shown a positive association between amount of free sugars consumption and dental caries experiences [31]. In China, per capita sugar production was continually increasing from 76.35 million tons in 2000 to 120.8 million tons in 2014, and added sugar food consumption was continually increasing from 8.2% in 2000 to 25.4% in 2009 [12].

Our study found the difference between urban and rural children, as well as the changing of the difference. DMF% and DMFT of 12-year-old Chinese children in urban group were higher than their rural counterparts from 1995 to 2010. However, over time, the gap between urban and rural areas gradually narrowed, and by 2014, the DMFT of rural children was even in line with that of urban children. One reason may be a convergence of urban and rural lifestyles has been occurring in China as urbanization advanced, resulting in an increase in caries-related unhealthy lifestyles among rural children [32], such as a growing obesity epidemic [33]. Besides, unequal distribution of oral health professionals and lack of appropriate health facilities mean that access to oral health services in rural areas is limited. As a result, rural children are becoming more vulnerable and should be taken seriously.

Also, our study found the difference between boys and girls. DMF% and DMFT in girls were higher than boys at all survey years. This result is consistent with a widely documented argument that girls have presented with a higher prevalence of dental caries than boys throughout time and across cultures [34, 35]. The reason is usually explained by the following three factors: (1) Earlier eruption of teeth in girls, hence longer exposure of girls’ teeth to the cariogenic oral environment [35]; (2) Girls prefer carbohydrates and sugary foods [36], which may lead to more dental caries experiences [35, 37]; (3) Physiological studies found that women's average salivary flow rate was significantly lower than men’s due to the effects of estrogen and saliva flow is the medium that brings protective agents into the oral cavity [38–40].

In terms of attendance to oral health services, we found that CFR levels of 12-year-old Chinese children from 1995 to 2014 were low and there was no gender difference, which is consistent with the results of the Fourth National Survey of Children’s Oral Health of China (15.6%) [29]. In Japan, 22.79% of students completing dental treatment in 2014 [41]. In New Zealand and Australia, the CFR even reached 90% [42]. This suggests that there is still a big gap in the treatment of children caries in China. It is unclear whether there is a gender difference in oral health service utilization and may be worth further study.

### Strengths and limitations

Our study has several strengths. First, the sample population is the largest and the best nationally representative student survey sample in China. Second, our study comprehensively assessed the development trends of dental caries in permanent teeth in 12-year-old Chinese children from 1995 to 2014, as well as the differences between urban–rural areas, boys and girls. These results commendably complement the research report and provide valuable reference for policy making in China and other middle-income countries. Meanwhile, our study also has several limitations that should be noted. First, we did not used lightweight portable examination light (in the blue-white color spectrum) used internationally to examine caries, and the dental caries examination was not performed in the dental chair, which may underestimate the prevalence of caries. However, the bias is within the acceptable range and studies using the same approach have been published in journals [43]. Therefore, the results across the five consecutive national surveys still comparable and can reflect the trend of caries in Chinese children. Second, we did not perform the calibration process for DMFT, and the inter-and intra-examiner Kappa statistics for DMFT. Although the use of different measurement instruments and different examiners could affect the consistency of the examination, they should not affect the assessment of trends of dental caries in permanent teeth over time, especially in the five survey cycles. The principles underlying the methods are the same ones and the quality control meets the requirements.

## Conclusions

In conclusion, our study found that, over the past 20 years, DMFT and DMF% of 12-year-old Chinese children presented V-shaped fluctuant upward trends with a decline trend from 1995 to 2000 and a significantly continuous upward trend from 2000 to 2014, and reached the lowest level in 2000. Meanwhile, caries filling situation had no significant improvement. The rural children and girls are the more vulnerable groups in the development of dental caries and need to pay priority. Our study supports the continuation of policies to improve students’ oral health.

## Supplementary Information

**Additional file 1: Table 1**. Secular changes of DMF%, DMFT and CFRamong 12-year-old Chinese children from 1995 to 2014.

**Additional file 2: Table 2**. The classification of DMF% and DMFT in 12-year-old Chinese children from 1995-2014 (%, 95%CI).

## Data Availability

The datasets used and/or analyzed during the current study are not publicly available but are available from the corresponding author on reasonable request.

## Abbreviations

DMF%: Prevalence of decayed, missing and filled teeth
DMFT: Mean decayed, missing, filled teeth score
CFR: Caries filling ratio
NCD: Non-communicable diseases
WHO: World Health Organization
CNSSCH: Chinese National Survey on Students’ Constitution and Health
DT: Decayed tooth
MT: Missing tooth
FT: Filled tooth
SPSS: Statistical Package for the Social Sciences
