# Supplementary material for: Trends of dental caries in permanent teeth among 12-year-old Chinese children: evidence from five consecutive national surveys between 1995 and 2014
Source: BMC Oral Health. 2021 Sep 23;21:467. doi: 10.1186/s12903-021-01814-7 (PMC8461869; doi:10.1186/s12903-021-01814-7)
Supplement: Supplementary file 1 — Additional file 1: Table 1. Secular changes of DMF%, DMFT and CFRamong 12-year-old Chinese children from 1995 to 2014. [file 12903_2021_1814_MOESM1_ESM.docx]

## Supplementary Table 1 Secular changes of DMF%, DMFT and CFR among 12-year-old Chinese children from 1995 to 2014

|  | 1995 | 2000 | 2005 | 2010 | 2014 | Absolute Change* | statistic | *P* Value |
| --- | --- | --- | --- | --- | --- | --- | --- | --- |
| **DMF%** |  |  |  |  |  |  |  |  |
| Total | 21.1 (20.5-21.7) | 15.9(15.3-16.4) | 16.2(15.7-16.8) | 21.9(21.3-22.5) | 24.3(23.6-24.9) | +3.2(2.3~4.0) | -13.124 | ＜0.001 |
| Area |  |  |  |  |  |  |  |  |
| Urban | 26.0(25.0-26.9) | 18.0(17.2-18.8) | 16.8(16.1-17.6) | 22.5(21.7-23.4) | 23.8(22.9-24.7) | -2.2(-3.4~-0.9) | -0.242 | 0.809 |
| Rural | 16.1(15.3-16.9)^#^ | 13.8(13.0-14.5)^#^ | 15.7(14.9-16.4)^#^ | 21.3(20.4-22.1)^#^ | 24.7(23.8-25.6) | +8.6(7.4~9.8) | -19.036 | ＜0.001 |
| Gender |  |  |  |  |  |  |  |  |
| Boys | 18.9(18.1-19.7) | 14.0(13.3-14.8) | 13.9(13.2-14.6) | 19.2(18.4-20.0) | 20.3(19.5-21.2) | +1.6(0.3~2.6) | -6.418 | ＜0.001 |
| Girls | 23.4(22.5-24.3)^#^ | 17.7(16.9-18.5)^#^ | 18.6(17.8-19.4)^#^ | 24.6(23.7-25.5)^#^ | 28.2(27.3-29.1)^#^ | +4.8(3.5~6.1) | -11.906 | ＜0.001 |
| Region |  |  |  |  |  |  |  |  |
| East | 27.4 (26.3-28.5)^a^ | 22.7 (21.8-23.7)^a^ | 20.0 (19.0-20.9)^a^ | 20.4 (19.4-21.4)^a^ | 28.6 (27.5-29.7)^a^ | +1.2(-0.3~2.7) | 1.551 | 0.121 |
| Central | 17.3 (16.2-18.3)^b^ | 9.7 (8.8-10.5)^b^ | 13.9 (12.9-14.9)^b^ | 20.7 (19.6-21.9)^b^ | 21.4 (20.2-22.6)^b^ | +4.1(2.6~5.7) | -1.746 | 0.081 |
| West | 16.9 (15.9-17.9)^b^ | 12.0 (11.1-12.8)^c^ | 14.0 (13.2-14.8)^b^ | 24.2 (23.2-25.3)^b^ | 22.0 (20.9-23.0)^b^ | +5.1(3.6~6.5) | -11.081 | ＜0.001 |
| **DMFT** |  |  |  |  |  |  |  |  |
| Total | 0.38±0.90 | 0.28±0.76 | 0.31±0.97 | 0.66±2.13 | 0.54±1.30 | 0.16±0.01 | 0.057 | ＜0.001 |
| Area |  |  |  |  |  |  |  |  |
| Urban | 0.48±1.00 | 0.31±0.78 | 0.32±1.02 | 0.81±2.65 | 0.54±1.27 | 0.06±0.01 | 0.035 | ＜0.001 |
| Rural | 0.29±0.78^#^ | 0.25±0.75^#^ | 0.29±0.92^#^ | 0.50±1.40^#^ | 0.54±1.33 | 0.25±0.01 | 0.077 | ＜0.001 |
| Gender |  |  |  |  |  |  |  |  |
| Boys | 0.33±0.81 | 0.23±0.68 | 0.25±0.84 | 0.57±1.95 | 0.42±1.08 | 0.09±0.01 | 0.039 | ＜0.001 |
| Girls | 0.44±0.98^#^ | 0.32±0.84^#^ | 0.36±1.09^#^ | 0.74±2.28^#^ | 0.66±1.48^#^ | 0.22±0.01 | 0.075 | ＜0.001 |
| Region |  |  |  |  |  |  |  |  |
| East | 0.52 (1.05)^a^ | 0.42 (0.95)^a^ | 0.39 (1.15)^a^ | 0.42 (1.03)^a^ | 0.69 (1.50)^a^ | 0.17±0.01 | 0.033 | ＜0.001 |
| Central | 0.30 (0.80)^b^ | 0.14 (0.49)^b^ | 0.26 (0.93)^b^ | 0.39 (0.99)^b^ | 0.46 (1.19)^b^ | 0.15±0.01 | 0.053 | ＜0.001 |
| West | 0.28 (0.75)^b^ | 0.20 (0.63)^c^ | 0.25 (0.77)^b^ | 1.09 (3.20)^b^ | 0.46 (1.13)^b^ | 0.17±0.01 | 0.128 | ＜0.001 |
| **CFR%** |  |  |  |  |  |  |  |  |
| Total | 17.4(16.5-18.3) | 22.8(21.7-24.0) | 19.3(18.3-20.4) | 23.4(22.6-24.2) | 15.6(14.9-16.3) | -1.8(-3.0~-0.7) | 1.927 | 0.054 |
| Area |  |  |  |  |  |  |  |  |
| Urban | 23.8(22.5-25.0) | 33.3(31.5-35.1) | 25.6(24.0-27.2) | 27.8(26.8-28.9) | 18.7(17.6-22.5) | -5.1(-6.7~-3.3) | 5.914 | ＜0.001 |
| Rural | 6.4(5.5-7.4)^#^ | 9.7(8.5-11.0)^#^ | 12.4(11.2-13.7)^#^ | 16.3(15.2-17.3)^#^ | 12.5(11.5-5.3)^#^ | +6.1(4.9~7.5) | -9.125 | ＜0.001 |
| Gender |  |  |  |  |  |  |  |  |
| Boys | 17.1(15.8-18.5) | 22.1(20.3-23.9) | 20.2(18.5-21.8) | 23.8(22.6-24.9) | 14.9(13.8-15.4) | -2.2(-4.0~-0.4) | 0.810 | 0.418 |
| Girls | 17.6(16.4-18.8) | 23.3(21.8-24.9) | 18.7(17.4-20.1) | 23.1(22.1-24.1) | 16.0(15.1-16.4) | -1.6(-3.1~-0.1) | 1.832 | 0.067 |
| Region |  |  |  |  |  |  |  |  |
| East | 23.4 (22.0-24.8)^a^ | 26.0 (24.5-27.6)^a^ | 26.8 (25.1-28.5)^a^ | 26.9 (25.2-28.5)^a^ | 23.1 (21.8-24.3)^a^ | -0.3(-2.2~1.5) | 0.534 | 0.593 |
| Central | 10.4 (8.9-11.9)^b^ | 17.8 (14.9-20.8)^b^ | 11.9 (10.0-13.8)^b^ | 10.6 (9.2-12.0)^b^ | 11.2 (9.8-12.5)^b^ | +0.8(-1.3~2.8) | 0.991 | 0.322 |
| West | 10.6 (9.0-12.1)^b^ | 16.8 (14.6-18.9)^b^ | 12.5 (10.9-14.1)^b^ | 25.4 (24.4-26.4)^a^ | 7.3 (6.3-8.2)^c^ | -3.3(-5.1~-1.5) | -2.724 | 0.006 |

Note: Indicators are presented as % (95%CI)

The statistic is Z value for DMF% and CFR and OR value for DMFT.

* DMF% (DMFT or CFR) in 2014 minus DMF% (DMFT or CFR) in 1995

^#^ *p* < 0.05

a, b, c: The same letters represent there was a difference between the group, and different letters represent there was no difference between the groups.
