# Supplementary material for: Trends of dental caries in permanent teeth among 12-year-old Chinese children: evidence from five consecutive national surveys between 1995 and 2014
Source: BMC Oral Health. 2021 Sep 23;21:467. doi: 10.1186/s12903-021-01814-7 (PMC8461869; doi:10.1186/s12903-021-01814-7)
Supplement: Supplementary file 2 — Additional file 2: Table 2. The classification of DMF% and DMFT in 12-year-old Chinese children from 1995-2014 (%, 95%CI). [file 12903_2021_1814_MOESM2_ESM.docx]

Supplementary Table 2 – The classification of DMF% and DMFT in 12-year-old Chinese children from 1995-2014 (%, 95%CI)

|  | 1995 | 2000 | 2005 | 2010 | 2014 |
| --- | --- | --- | --- | --- | --- |
| DMF% |  |  |  |  |  |
| DT | 17.9 (17.3-18.4) | 12.7 (12.2-13.2) | 13.4 (12.9-13.9) | 18.4 (17.8-19.0) | 20.4 (19.8-20.9) |
| MT | 0.7 (0.6-0.8) | 0.6 (0.5-0.7) | 0.6 (0.4-0.7) | 3.2 (3.0-3.5) | 1.2 (1.1-1.4) |
| FT | 4.1 (3.8-4.4) | 3.9 (3.6-4.2) | 3.3 (3.1-3.6) | 5.8 (5.5-6.2) | 4.6 (4.3-5.0) |
| DMFT |  |  |  |  |  |
| DT | 0.31 (0.30-0.32) | 0.21 (0.20-0.22) | 0.24 (0.23-0.25) | 0.40 (0.38-0.41) | 0.43 (0.41-0.45) |
| MT | 0.01 (0.01-0.01) | 0.01 (0.01-0.01) | 0.01 (0.01-0.01) | 0.11 (0.09-0.12) | 0.03 (0.02-0.03) |
| FT | 0.07 (0.06-0.07) | 0.06 (0.06-0.07) | 0.06 (0.05-0.06) | 0.15 (0.14-0.17) | 0.08 (0.08-0.09) |
